# Second-line HIV treatment failure in sub-Saharan Africa: A systematic review and meta-analysis

**DOI:** 10.1371/journal.pone.0220159

**Published:** 2019-07-29

**Authors:** Dumessa Edessa, Mekonnen Sisay, Fekede Asefa

**Affiliations:** 1 Department of Clinical Pharmacy, School of Pharmacy, College of Health and Medical Sciences, Haramaya University, Harar, Oromia, Ethiopia; 2 Department of Pharmacology and Toxicology, School of Pharmacy, College of Health and Medical Sciences, Haramaya University, Harar, Oromia, Ethiopia; 3 School of Public Health, College of Health and Medical Sciences, Haramaya University, Harar, Oromia, Ethiopia; 4 Center for Midwifery, Child and Family Health, Faculty of Health, University of Technology Sydney, NSW, Australia; University of Cincinnati College of Medicine, UNITED STATES

## Abstract

**Background:**

Increased second-line antiretroviral therapy (ART) failure rate narrows future options for HIV/AIDS treatment. It has critical implications in resource-limited settings; including sub-Saharan Africa (SSA) where the burden of HIV-infection is immense. Hence, pooled estimate for second-line HIV treatment failure is relevant to suggest valid recommendations that optimize ART outcomes in SSA.

**Methods:**

We retrieved literature systematically from PUBMED/MEDLINE, EMBASE, CINAHL, Google Scholar, and AJOL. The retrieved studies were screened and assessed for eligibility. We also assessed the eligible studies for their methodological quality using the Joanna Briggs Institute’s appraisal checklist. The pooled estimates for second-line HIV treatment failure and its associated factors were determined using STATA, version 15.0 and MEDCALC, version 18.11.3, respectively. We assessed publication bias using Comprehensive Meta-analysis software, version 3. Detailed study protocol for this review/meta-analysis is registered and found on PROSPERO (ID: CRD42018118959).

**Results:**

A total of 33 studies with the overall 18,550 participants and 19,988.45 person-years (PYs) of follow-up were included in the review. The pooled second-line HIV treatment failure rate was 15.0 per 100 PYs (95% CI: 13.0–18.0). It was slightly higher at 12–18 months of follow-up (19.0/100 PYs; 95% CI: 15.0–22.0), in children (19.0/100 PYs; 95% CI: 14.0–23.0) and in southern SSA (18.0/100 PYs; 95% CI: 14.0–23.0). Baseline values (high viral load (OR: 5.67; 95% CI: 13.40–9.45); advanced clinical stage (OR: 3.27; 95% CI: 2.07–5.19); and low CD4 counts (OR: 2.80; 95% CI: 1.83–4.29)) and suboptimal adherence to therapy (OR: 1.92; 95% CI: 1.28–2.86) were the factors associated with increased failure rates.

**Conclusion:**

Second-line HIV treatment failure has become highly prevalent in SSA with alarming rates during the 12–18 month period of treatment start; in children; and southern SSA. Therefore, the second-line HIV treatment approach in SSA should critically consider excellent adherence to therapy, aggressive viral load suppression, and rapid immune recovery.

## Introduction

In the past decade, rapid scale-up of antiretroviral therapy (ART) in sub-Saharan Africa (SSA) substantially reduced HIV/AIDS-related morbidity and mortality [1, 2]. It has also prolonged the average life expectancy of HIV-infected individuals [1]. However, these benefits are being challenged by the increasing HIV treatment failure rates with first or second-line antiretroviral therapies [3–6]. HIV treatment failure can be defined in terms of clinical, immunological, or virological failures [7]. Clinical failure is the occurrence of a new or recurrent stage III or stage IV clinical event (s). Immunological failure is the decline of CD4 counts either to less than the pre-treatment value or to <50% of a peak value on ART or persistently lower than 100 cells/ml [7]. Virological failure could be a definite failure (i.e., when a single viral load (VL) is greater than 10,000 copies/ml at 12 months of follow-up) or a probable failure (i.e., when either a single VL is >1000 copies/ml at 12 months or a VL at 12 months is ≥ 400 copies/ml confirmed by a second measurement taken 30 days later) [8–10]. Clinical and immunological failure criteria are not sufficient for the definite diagnosis of treatment failure and each of them should be accompanied with VL tests as a confirmation [11]. With this, VL testing is efficient to indicate direct plasma effects of ART on HIV ribonucleic acid (RNA) [12]. It also helps preserve the limited HIV treatment options available by reducing the probability of incorrect switching to the next more expensive and toxic regimens [13].

The HIV-infected patients on ART are recalling earlier fears of death from the infection because of treatment failures [14]. Patients who experienced first-line HIV treatment failure may be switched to second-line regimens [15, 16]. Many countries in resource-limited settings switch a failed first-line ART to second-line regimen after an initial delay mainly related to inadequate VL tests [11]. The inadequacy of VL informed differentiated care for the HIV-infected patients commenced with second-line ART [17] could increase the risks of death and opportunistic infections especially in patients with advanced HIV at the time of first-line HIV treatment failure [18].

The HIV treatment failure involving second-line regimens has very narrow options for further switching, and this is a serious concern in resource-limited settings [19]. The World Health Organization (WHO) recommends few second-line regimens as preferred ART (i.e., ritonavir-boosted atazanavir- or lopinavir-based ART and dolutegravir-based ART) [20, 21]. Despite the limited second-line HIV treatment options, many countries in SSA have financial constraints to adopt third-line regimens [20, 21]. As a result, the optimal use of second-line therapies after the occurrence of first-line HIV treatment failure is alarmingly essential for SSA, the epicenter of HIV/AIDS. However, many countries in SSA have no national strategic guidelines for the optimal use of second-line therapy despite the occurrence of a number of treatment failures related to the therapies [17].

Suboptimal adherence (i.e., missing of any dose in the past 3 days [22] or 7 days [23]; or less than 95% adherence in the past 30 days [24] or less than 90% adherence in the past year [25]) was indicated as a key determinant of second-line HIV treatment failure [26, 27]. Suboptimal adherence could be a result of regimen toxicities [28]. It may require a tailored adherence intervention based on the degree of suboptimal adherence [27, 29]. Baseline characteristics such as delayed initiation of second-line therapy [30] and high VL might result in unfavorable treatment outcomes [25]. To maximize the durability of the second-line regimens, early identification of first-line treatment failure and switching to a second-line regimen at a relatively higher CD4 cell count is very important [31, 32]. Advanced clinical stage at baseline and lack of VL monitoring were also identified to have associations with second-line HIV treatment failures [33]. In addition, the clinical status of patients such as baseline clinical stage IV and CD4 counts below 100 cells/mm3 were significantly linked with increased rates of treatment failure [34]. As a result, pooling the proportion of second-line HIV treatment failures and factors associated with these failures are required to assist the optimization of HIV treatment outcomes in SSA. Therefore, the aim of this systematic review and meta-analysis was to estimate the proportion of second-line HIV treatment failure and its associated factors in SSA.

## Materials and methods

### Study protocol

The method of this systematic review and meta-analysis was reported as per the Preferred Reporting Items for Systematic Review and Meta-Analysis Protocols (PRISMA-P) 2015 statement recommendations [35]. Identification of records, screening by titles and abstracts, and eligibility evaluation of full texts for final inclusion was conducted in accordance with the PRISMA flow diagram [36]. During the execution of this systematic review and meta-analysis, the PRISMA checklist was strictly followed. The protocol is registered on the International Prospective Register of Systematic Reviews (PROSPERO) with a registration number of CRD42018118959 and it is available at https://www.crd.york.ac.uk/prospero/#recordDetails.php?ID=CRD42018118959 [37].

### Data sources and searches

We performed a systematic literature search from PubMed, MEDLINE (Ovid), EMBASE (Ovid), CINAHL (EBSCOhost), Cochrane Library, Google Scholar, Health Technology Assessment, African Journals Online (AJOL) and ResearchGate. Websites of organizations and University repositories were also visited to retrieve any remaining relevant record including unpublished (gray) kinds of literature. In our search strategy, search terms we employed were “treatment failure”; “second-line”; “protease inhibitor”; “antiretroviral therapy”; and names of countries in the SSA. During the search, we accomplished a careful selection of keywords and indexing terms that did not limit the year of publication. In the search strategy, Boolean operators and truncations were also employed. The search was conducted from 15 December 2018 to 14 January 2019. Accordingly, all published and unpublished literature identified during the period of searching were retrieved.

### Study selection

We set predefined inclusion and exclusion criteria for initial screening by titles or abstracts and evaluation of full texts for their eligibility assessment. We considered articles with at least an outcome of failure to second-line ART for their potential to be included. Next, we assessed the original articles reporting second-line HIV treatment failure after at least 6 month period of follow-up; reported in English language; and conducted in countries of SSA for their eligibility. In addition, we assessed the eligible original articles for quality using the Joanna Briggs Institute’s (JBI) critical appraisal checklist and articles with moderate (50–75%) to high (>75%) quality were considered as per the appraisers’ evaluation results. However, we excluded articles with outcomes not related to second-line therapy failure; with no separate failure data for SSA patients in case of mixed multi-center study settings involving SSA and other countries; and with no separate data of second-line therapy failure in studies involving first and second-line therapies during the screening and eligibility assessments.

### Screening and eligibility

We identified and selected records retrieved through a search of the electronic databases and indexing services. Following this, we exported them to ENDNOTE reference software version 8.2 (Thomson Reuters, Stamford, CT, USA). Next, we identified, registered, and removed duplicates by the use of ENDNOTE. Accordingly, two authors, Dumessa Edessa (DE) and Mekonnen Sisay (MS), independently screened titles and abstracts of the retained records based on the predefined inclusion criteria. A third author, Fekede Asefa (FA), was consulted in case of disagreement between the two authors. With this, DE and FA individually collected and evaluated full texts of the retained articles for their quality and final eligibility assessment. In the end, we included articles that fulfilled the quality evaluation criteria.

### Quality assessment and data extraction

We accomplished quality assessment for the articles by employing the JBI’s critical appraisal checklist for cohort and analytical cross-sectional studies [38, 39]. Two authors (DE and MS) critically appraised the articles. For the final decision of inclusion, we considered scores of the two authors in consultation with the third author’s score (in case of disagreement between the two authors’ appraisal results). Lastly, we ranked the articles by their methodological qualities based on the total number of appraisers’ score marked as ‘yes’ to questions of the JBI’s critical appraisal checklist. Accordingly, we included all studies with their overall positive responses in ranges of 50% to 75% (moderate quality studies) or higher than 75% (high quality studies) for the review and meta-analysis.

To extract relevant data, we employed a customized data abstraction format that has been prepared in a Microsoft Excel sheet. Two of the authors independently abstracted data pertaining to first author; year of publication; study design (analytical cross-sectional, follow-up); study region/country; study participants (children, adults, mixed-age groups); types of second-line ART (ritonavir-boosted protease inhibitor (PI)-based ART, PI-based ART with no ritonavir-boosting); sample size; median months of follow-up; person/patient-years (PYs) of follow-up; and event of interest (number of second-line therapy failure and factors associated with the failure).

### Outcome variables

Proportion of second-line HIV treatment failure that includes clinical, immunological or virological failure, as defined by the WHO [7, 9] was the primary outcome variable we estimated in this systematic review and meta-analysis. The secondary outcome measure we estimated was factors associated with the second-line HIV treatment failures.

### Data synthesis and analysis

Proportion of second-line HIV treatment failure we pooled together was accomplished with the help of STATA software, version 15.0. Again, we performed sensitivity and subgroup analyses to minimize the risks of bias. With this, we used forest plots to graphically report the various meta-analysis results. We also applied the Mantel-Haenszel random-effects model to conduct meta-analyses at a 95% confidence level. Likewise, we assessed the heterogeneity status of the included studies and presented it with the use of Cochran’s Q test (chi-squared (I^2^) statistic). We also accomplished tests for factors associated with second-line therapy failure by using MEDCALC statistical software (MedCalc Software bvba, Ostend, Belgium), version 18.11.3. Besides, we employed Comprehensive Meta-analysis software (Biostat, Englewood, New Jersey, USA), version 3, for publication bias assessment. Similarly, we evaluated the presence of publication bias with the use of Egger's regression and Begg’s correlation tests. Lastly, we considered all statistical tests with p-values less than 0.05 (two-tailed) as significant.

## Results

**Fig 1:**
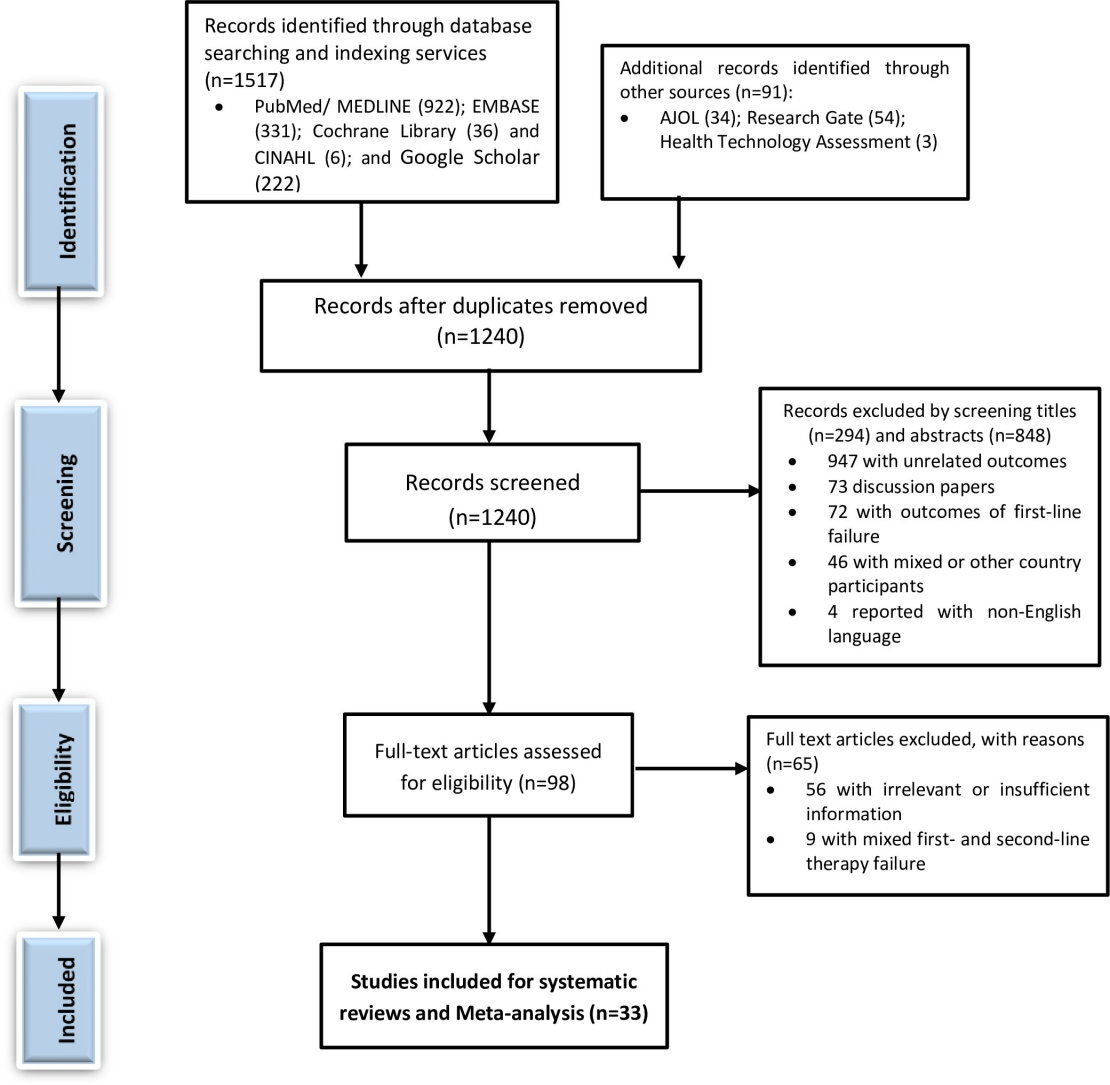
PRISMA flow diagram depicting the selection process.

We identified a total of 1,608 records from the search of legitimate databases and indexing services. After the removal of 368 duplicates, we retained 1240 records for screening by titles and abstracts. Again, we excluded a total of 1,142 literature by screening titles (n = 294) and abstracts (n = 848). Of this 1,142 literature, 947 of them had unrelated outcomes of interest; 73 of them were discussion papers; 72 of them had outcomes of first-line therapy failure; 46 of them had mixed and/or other country studies, and 4 of them reported their outcomes with non-English languages. Accordingly, we conducted an eligibility evaluation of 98 full texts as per the predefined eligibility criteria for inclusion. Again, we excluded 65 studies with justifiable reasons (i.e., 56 of them with irrelevant/insufficient outcomes of interest; 9 of them with mixed first and second-line HIV treatment failures and no separate data for second-line therapy failure). PRISMA flow chart depicting the selection, screening, and eligibility assessment process is shown in Fig 1 and S1 Table. We also assessed these records for their methodological quality by employing the JBI’s critical appraisal checklists (Table 1). Finally, we included 33 articles with the primary outcome of interest and with a high or moderate percentage in its score of methodological quality assessment for the systematic reviews and meta-analysis.

**Table 1:** Quality assessment for included studies.

References	JBI’s Critical Appraisal Checklist										
	Q1	Q2	Q3	Q4	Q5	Q6	Q7	Q8	Q9	Q10	Q11
Adetunji et al, 2013	NA	NA	Yes	Yes	Yes	Yes	Yes	Yes	UC	No	Yes
Akanmu et al, 2015	NA	NA	Yes	Yes	Yes	Yes	Yes	Yes	UC	No	Yes
Berhanu et al, 2014	NA	NA	Yes	No	No	Yes	Yes	Yes	Yes	No	Yes
Boender et al, 2016	NA	NA	Yes	Yes	Yes	Yes	Yes	Yes	Yes	Yes	Yes
Boerma et al, 2017	NA	NA	Yes	No	No	Yes	Yes	Yes	Yes	No	Yes
Castelnuovo et al, 2009	NA	NA	Yes	No	No	Yes	Yes	Yes	Yes	No	Yes
Ciaffi et al, 2015	NA	NA	Yes	Yes	Yes	Yes	Yes	Yes	Yes	Yes	Yes
Collier et al, 2017	NA	NA	Yes	Yes	Yes	Yes	Yes	Yes	Yes	No	Yes
Court et al, 2014	NA	NA	Yes	Yes	Yes	Yes	Yes	Yes	No	No	Yes
Evans et al, 2018	NA	NA	Yes	Yes	Yes	No	Yes	Yes	No	No	Yes
Evans et al, 2018	NA	NA	Yes	Yes	Yes	No	Yes	Yes	No	No	Yes
Fox et al, 2010	NA	NA	Yes	Yes	Yes	Yes	Yes	Yes	Yes	No	Yes
Fox et al, 2016	NA	NA	Yes	No	No	Yes	Yes	Yes	Yes	No	Yes
Garone et al, 2013	NA	NA	Yes	Yes	Yes	Yes	Yes	Yes	Yes	Yes	Yes
Hosseinipour et al, 2010	NA	NA	Yes	Yes	Yes	Yes	Yes	Yes	Yes	No	Yes
Johnston et al, 2012	NA	NA	Yes	Yes	Yes	Yes	Yes	Yes	Yes	No	Yes
Johnston et al, 2014	NA	NA	Yes	Yes	Yes	Yes	Yes	Yes	Yes	No	Yes
Levison et al, 2012	NA	NA	Yes	No	No	Yes	Yes	Yes	Yes	No	Yes
Murphy et al, 2012	NA	NA	Yes	Yes	Yes	Yes	Yes	Yes	Yes	No	Yes
Musiime et al, 2013	NA	NA	Yes	Yes	Yes	Yes	Yes	Yes	Yes	No	Yes
Ongubo et al, 2017	NA	NA	Yes	Yes	Yes	Yes	Yes	Yes	No	No	Yes
Onyedum et al, 2013	NA	NA	Yes	No	No	Yes	Yes	Yes	Yes	No	Yes
Paton et al, 2014	NA	NA	Yes	Yes	Yes	Yes	Yes	Yes	Yes	Yes	Yes
Paton et al, 2017	NA	NA	Yes	Yes	Yes	Yes	Yes	Yes	Yes	Yes	Yes
Pujades et al, 2010	NA	NA	Yes	Yes	Yes	Yes	Yes	Yes	Yes	No	Yes
Rawizza et al, 2013	NA	NA	Yes	No	No	Yes	Yes	Yes	Yes	Yes	Yes
Schoffelen et al, 2013	NA	NA	Yes	Yes	Yes	Yes	Yes	Yes	Yes	No	Yes
Shearer et al, 2017	NA	NA	Yes	Yes	Yes	Yes	Yes	Yes	Yes	No	Yes
Sigaloff et al, 2012	NA	NA	Yes	Yes	Yes	Yes	Yes	Yes	Yes	No	Yes
Tsegaye et al, 2016	NA	NA	Yes	Yes	Yes	Yes	Yes	Yes	Yes	No	Yes
Wandeler et al, 2012	NA	NA	Yes	Yes	Yes	Yes	Yes	Yes	Yes	No	Yes
Wandeler et al, 2014	NA	NA	Yes	Yes	Yes	Yes	Yes	Yes	Yes	No	Yes
Van Zyl et al, 2011	Yes	Yes	Yes	Yes	Yes	No	Yes	Yes			

**Note**: NA, not applicable; UN, unclear; Q1-8, JBI’s Critical Appraisal Checklist for Analytical Cross Sectional studies {Q1: Were the criteria for inclusion in the sample clearly defined? Q2: Were the study subjects and the setting described in detail? Q3: Was the exposure measured in a valid and reliable way? Q4: Were objective, standard criteria used for measurement of the condition? Q5: Were confounding factors identified? Q6: Were strategies to deal with confounding factors stated? Q7: Were the outcomes measured in a valid and reliable way? Q8: Was appropriate statistical analysis used?}; Q1-11, JBI’s Critical Appraisal Checklist for Cohort studies {Q1: Were the two groups similar and recruited from the same population? Q2: Were the exposures measured similarly to assign people to both exposed and unexposed groups? Q3: Was the exposure measured in a valid and reliable way? Q4: Were confounding factors identified? Q5: Were strategies to deal with confounding factors stated? Q6: Were the groups/participants free of the outcome at the start of the study (or at the moment of exposure)? Q7: Were the outcomes measured in a valid and reliable way? Q8: Was the follow up time reported and sufficient to be long enough for outcomes to occur? Q9: Was follow up complete, and if not, were the reasons to loss to follow up described and explored? Q10: Were strategies to address incomplete follow up utilized? Q11: Was appropriate statistical analysis used?}.

### Study characteristics

The 33 studies included for the systematic reviews and meta-analysis had a total of 18,550 participants, and 2,473 of them experienced treatment failures to their second-line HIV treatment. The study participants had a total of 19,988.45 PYs of follow-up. All the included studies were published during the year 2009 to 2018. Sample sizes for the included studies range from 40 patients enrolled by a study conducted in Uganda [40] to 6,714 patients enrolled by a study accomplished in Nigeria [41]. The study participants of 26 studies were adults [19, 23–25, 27, 34, 40, 42–60], while that of 2 studies [22, 61] and 5 studies [6, 41, 62–64] were children and mixed age groups, respectively. Sixteen studies (n = 16) were from southern Africa [23, 27, 43, 45–52, 57, 58, 60, 62, 64]; 7 studies were from eastern Africa [22, 34, 40, 53, 55, 61, 63]; 5 studies were from western Africa [25, 41, 42, 44, 54]; and 5 studies were from mixed regions in SSA [6, 19, 24, 55, 59]. The second-line HIV treatment regimens received by the study participants were PI-based, 18 of them with ritonavir-boosted PI-based ART [24, 25, 27, 40, 42, 44, 45, 48, 49, 52–56, 58, 60, 61, 63] and 15 of them with no ritonavir in their PI-based ART regimens [6, 19, 22, 23, 34, 41, 43, 46, 47, 50, 51, 57, 59, 62, 64]. Thirteen studies (n = 13) defined the second-line HIV treatment failure by using the WHO definition of RNA VL more than 400 copies/ml [6, 19, 24, 25, 27, 42, 47, 49, 53, 54, 56–58] while 16 of the studies employed the WHO criteria of HIV RNA VL above 1000 copies/ml [22, 23, 40, 43–46, 48, 50–52, 55, 59–61, 63]. However, 4 of the studies employed mixed definitions of the WHO criteria for ART failure that included clinical, immunological and virological failures and/or death/lost to follow-up [34, 41, 62, 64] (Table 2 and S2 Table).

**Table 2:** Characteristics of studies describing second-line ART failure among patients on treatment follow-up in sub-Saharan Africa.

References	Year of publication	Study design	Study setting	Patient groups	Second-line regimen	Sample size	Number with TF	PYs of follow-up
Adetunji et al [25]	2013	RFU	Nigeria	Adults	PI/r-based	225	34	225
Akanmu et al [42]	2015	RFU	Nigeria	Adults	LPV/r-based	318	25	636
Berhanu et al [43]	2014	RFU	South Africa	Adults	PI-based	372	129	465
Boender et al [19]	2016	FU	Zambia, South Africa, Kenya, Uganda, Zimbabwe and Nigeria	Adults	PI-based	227	32	227
Boerma et al [22]	2017	FU	Uganda	Children	PI-based	60	12	120
Castelnuovo et al [40]	2009	FU	Uganda	Adults	LPV/r-based	40	7	120
Ciaffi et al [44]	2015	FU	Cameroon, Senegal and Burkina Faso	Adults	PI/r-based	451	5	451
Collier et al [45]	2017	FU	South Africa	Adults	LPV/r-based	101	23	202
Court et al [46]	2014	RFU	South Africa	Adults	PI-based	228	26	228
Evans et al [23]	2018	RFU	South Africa	Adults	PI-based	128	50	192
Evans et al [47]	2018	RFU	South Africa	Adults	PI-based	719	36	1438
Fox et al [49]	2010	FU	South Africa	Adults	PI-based	262	59	262
Fox et al [48]	2016	FU	South Africa	Adults	LPV/r-based	388	106	446.6
Garone et al [62]	2013	FU	South Africa	Mixed-age groups	PI-based	40	7	30
Hosseinipour et al [63]	2010	FU	Malawi	Mixed-age groups	LPV/r-based	101	15	101
Johnston et al [50]	2014	FU	South Africa	Adults	PI-based	122	39	518.75
Johnston et al [51]	2012	FU	South Africa	Adults	LPV/r-based	417	43	152.5
Levison et al [52]	2012	RFU	South Africa	Adults	LPV/r-based	322	43	268.3
Murphy et al [27]	2012	FU	South Africa	Adults	LPV/r-based	136	26	136
Musiime et al [61]	2013	FU	Uganda	Children	LPV/r-based	142	55	142
Ongubo et al [53]	2017	RFU	Malawi	Adults	ATV/r-based	376	35	282
Onyedum et al [54]	2013	RFU	Nigeria	Adults	LPV/r-based	68	12	68
Paton et al [55]	2014	FU	Five countries in SSA	Adults	LPV/r-based	379	35	758
Paton et al [56]	2017	FU	Malawi, Uganda, Zimbabwe and Kenya	Adults	LPV/r-based	336	45	1008
Pujades et al [6]	2010	FU	Burkina Faso, Democratic Republic of Congo, Kenya, Malawi, Mozambique, Nigeria, Zimbabwe, South Africa, Uganda, Zambia	Mixed-age groups	PI-based	493	91	493
Rawizza et al [41]	2013	RFU	Nigeria	Mixed-age groups	PI-based	6714	673	3357
Schoffelen et al [64]	2013	RFU	South Africa	Mixed-age groups	PI-based	191	48	318.3
Shearer et al [57]	2017	RFU	South Africa	Adults	PI-based	927	233	927
Sigaloff et al [24]	2012	FU	Uganda, South Africa, Kenya, Nigeria, Zambia and Zimbabwe	Adults	PI/r-based	232	63	232
Tsegaye et al [34]	2016	RFU	Ethiopia	Adults	PI-based	356	67	712
Van Zyl et al [58]	2011	CS	South Africa	Adults	LPV/r-based	93	37	93
Wandeler et al [59]	2014	FU	South Africa, Zambia, Zimbabwe	Adults	PI-based	1256	122	3495
Wandeler et al [60]	2012	FU	Zambia and South Africa	Adults	LPV/r-based	2330	240	1884
**Total**						**18, 550**	**2, 473**	**19, 988.45**

**Note**: CS, cross sectional; FU, follow-up; RFU, retrospective follow-up; ATV/r, ritonavir-boosted atazanavir; PI/r, ritonavir-boosted protease inhibitor; LPV/r, ritonavir-boosted lopinavir; PI, protease inhibitor; PYs, Person-years of follow-up; TF, treatment failure; SSA, sub-Saharan Africa.

### Proportion of patients with second-line ART failure

**Fig 2:**
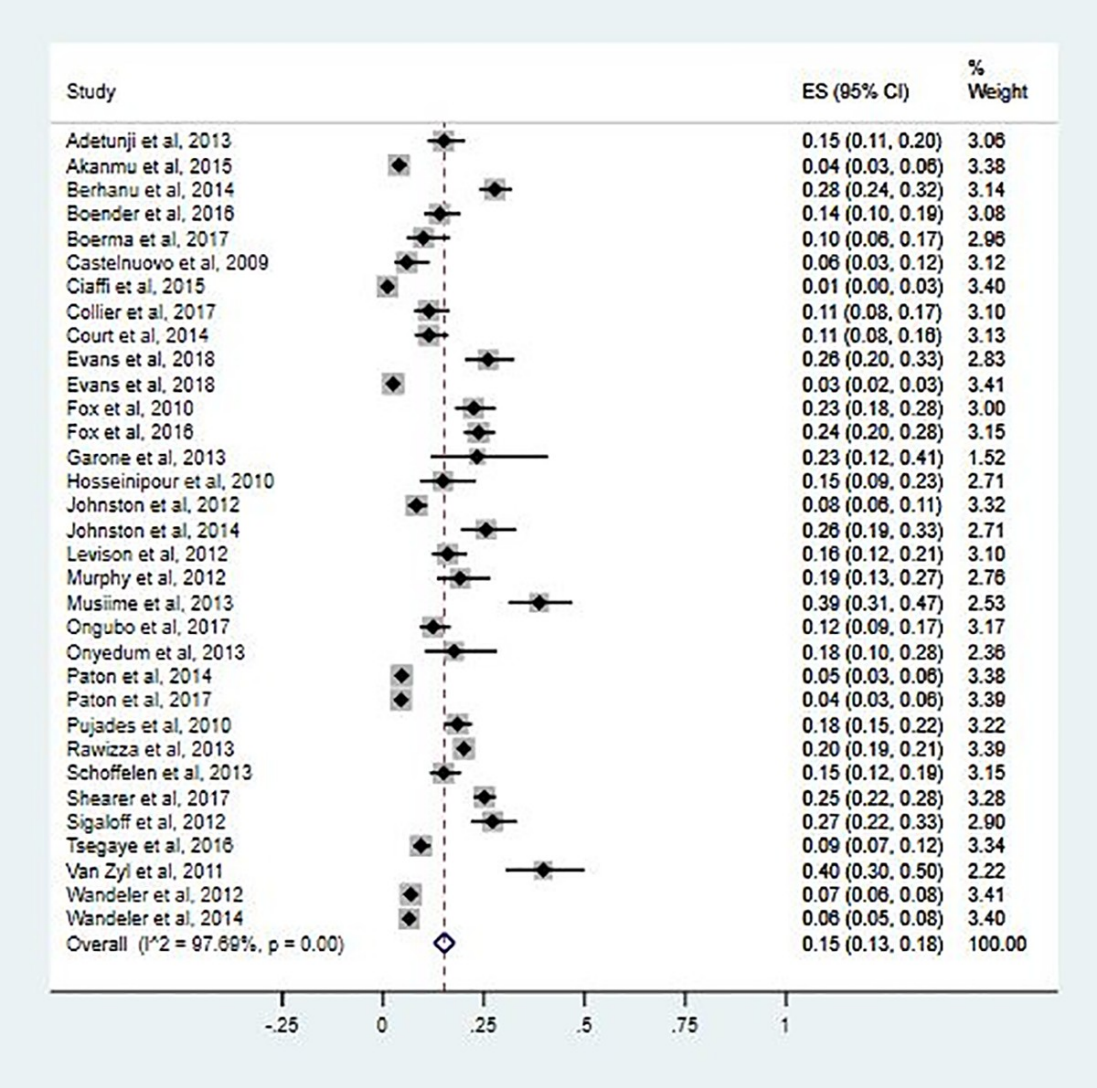
Forest pilot of proportion for second-line HIV treatment failure in SSA.

The pooled estimate for rate of second-line HIV treatment failure was 15.0 per 100 PYs of follow-up (95% CI: 13.0–18.0 per 100 PYs; I^2^ = 97.69%; P<0.001). The second-line treatment failures among the included studies range from 1.0/100 PYs (95% CI: 0.0–3.0 per 100 PYs) to 40.0/100 PYs (95% CI: 30.0–50.0) (Fig 2).

### Sensitivity and subgroup analyses

**Fig 3:**
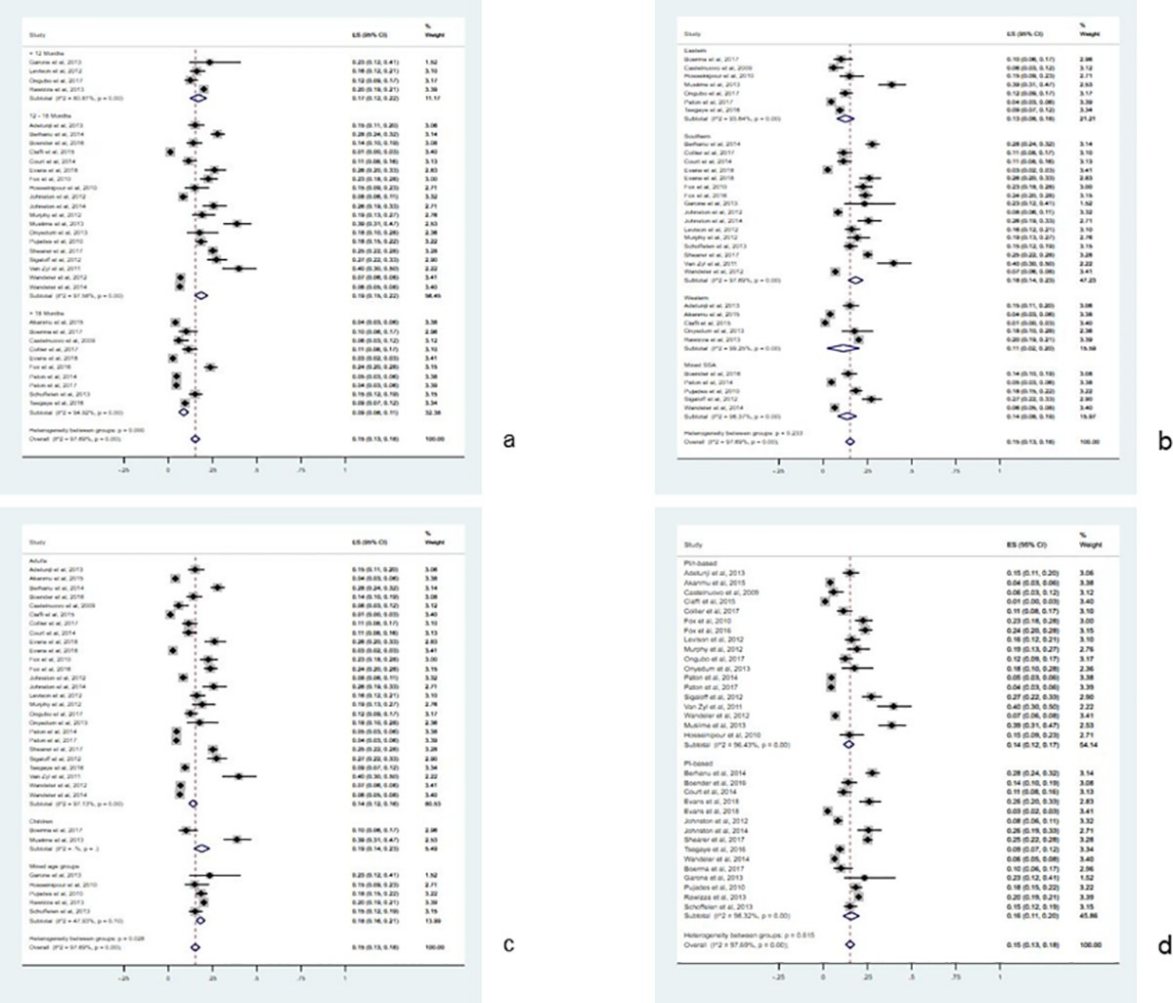
Forest pilots of proportion for second-line HIV treatment failure by subgroups. (a) Forest pilot describing failure by months of follow-up. (b) Forest pilot describing failure by regions of SSA. (c) Forest pilot describing failure by age group of participants. (d) Forest pilot describing failure by ritonavir boosting status of PI-based ART.

We performed sensitivity analyses by excluding outliers [44, 58] and one or more studies. They did not have significant changes in the extent of pooled outcome measures. As a result, we included all the studies for the meta-analysis. We performed subgroup analyses on the basis of month period of follow-up after second-line ART initiation (less than 12 months, 12–18 months, above 18 months); patient groups (children, adults, mixed age-groups); regions in SSA (southern Africa, eastern Africa, western Africa, mixed regions of SSA); and type of second-line ART (PI-based ART, ritonavir-boosted PI-based ART). Accordingly, the pooled estimates of second-line ART failure were 19.0/100 PYs (95% CI: 15.0–22.0/100 PYs; I^2^ = 97.58%; P<0.001) at 12–18 month period of follow-up after second-line therapy initiation; 19.0/100PYs (95% CI: 14.0–23.0/100PYs; I^2^ = 0.0%) among children; and 18.0/100 PYs (95% CI: 14.0–23.0/100 PYs; I^2^ = 97.60%; P < 0.001) among patients in the southern SSA (Fig 3A–3D and S3 Table).

### Factors associated with second-line ART failure

The pooled estimate for factors associated with second-line HIV treatment failure revealed that certain factors were influencing the failure rates. High baseline viral load (OR: 5.67; 95% CI: 13.40–9.45); advanced clinical stage of HIV at baseline (OR: 3.27; 95% CI: 2.07–5.19); low peak CD4 cell counts at baseline (<100 cells/mm3) (OR: 2.80; 95% CI: 1.83–4.29); and suboptimal adherence to second-line therapy (OR: 1.92; 95% CI: 1.28–2.86) were patient factors associated with the significantly increased occurrence of second-line ART failures (Table 3).

**Table 3:** Pooled estimates of factors associated with second-line HIV treatment failure.

Factor	OR (95% CI)	Z statistic	P-values
High VL at second-line therapy initiation	5.67 (3.40–9.45)	6.67	<0.0001
Advanced WHO clinical stage at baseline	3.27 (2.07–5.19)	5.06	<0.0001
Low CD4 cell counts (<100 cells/mm3) at baseline	2.80 (1.83–4.29)	4.75	<0.0001
Suboptimal adherence to second-line ART	1.92 (1.28–2.86)	3.20	0.0013

**Note**: VL, viral load; OR, odds ratio; ART, antiretroviral therapy; WHO, World Health Organization.

Different independent study reports also described depressive symptoms [23]; tuberculosis co-treatment with HIV/AIDS [45]; traditional medicine use [23]; delayed second-line HIV treatment initiation [25]; and younger age [53] as factors that favored second-line therapy failure. On the other hand, a study report indicated obesity [53] and elevated total bilirubin [53] as factors that protected second-line ART failure.

### Publication bias

**Fig 4:**
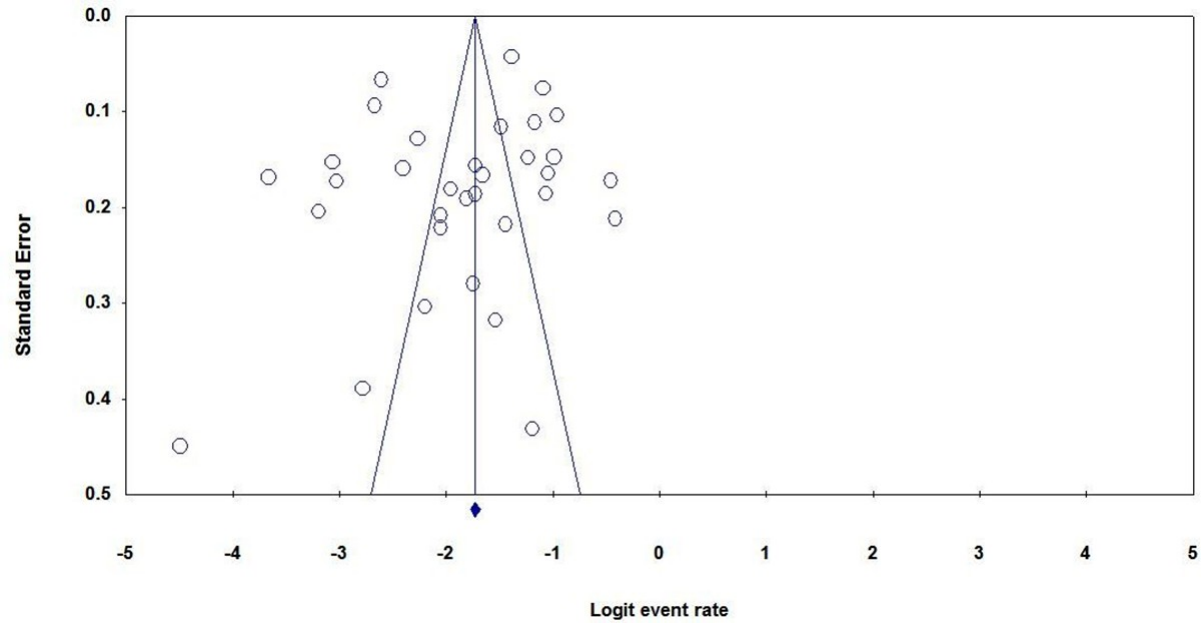
Funnel plot of standard error by logit event rate for publication bias.

Egger’s regression test did not show any evidence for publication bias among the included studies (P = 0.0992, one-tailed). In addition, Begg’s correlation test did not also show any evidence of publication bias (P = 0.154, one-tailed) (Fig 4).

## Discussion

In this meta-analysis, the pooled estimate of proportion for second-line HIV treatment failure was 15.0 per 100 PYs of follow-up. This evidence is in line with reports from several studies that revealed a proportion of second-line HIV treatment failure as high as 25% [32, 65–69]. With this, the rate of second-line therapy failure among HIV-infected children was estimated to be 19.0 per 100 PYs. Aligned with this finding, 19% of children treated with PI-based second-line therapy in Thailand encountered treatment failures [70]. Another study conducted in Thailand also reported up to 49% virological failure rates in children treated with second-line therapies [71]. Additionally, an ART audit for pediatric patients in London indicated that 37% of the patients achieved HIV-RNA VL less than 400 copies/ml [72]. Lack of VL monitoring, insufficient early diagnosis of failure, and unstructured and inadequate adherence counseling were the few reasons implicated for the increased treatment failure rates in children [73].

The pooled treatment failure rates before 12 months and 12–18 months of follow-up after second-line therapy initiation were 17.0/100 PYs and 19.0/100 PYs, respectively. The failure rate was 9.0/100 PYs after 18 months of follow-up. This indicated that a relatively sustained virological response is expected after the 18 months of follow-up. Similarly, a multi-centered study in Asia and Africa found that the most frequent experience of second-line therapy failure (i.e., 250.0/1000 PYs) occurred during 6 to 11 months of follow-up compared to the 18 months and more duration of follow-up (i.e., 212.0/1000 PYs) [6].

Subgroup analyses by regions revealed lower second-line HIV treatment failure rates in western (11.0/100 PYs) and eastern (13.0/100 PYs) regions of SSA compared to the rate in southern (18.0/100 PYs) region. These estimates are in line with the 19.6 million people living with HIV in southern and eastern regions compared to the 6.1 million people living with the infection in western and central Africa regions in 2017 [74]. Since a minimal failure rate can naturally occur toward antimicrobial agents, an increased probability of failure might be expected in the southern/eastern regions of Africa with a higher HIV burden. Indeed, in the presence of infection and antimicrobial agent use, there is always a natural phenomenon of drug resistance and failure [75]. This phenomenon can also be accelerated with improper infection control practices and suboptimal adherence to the ART [75]. In addition, two studies indicated consistent findings with the second-line HIV treatment failure rates in southern (19%) [76] and western/eastern regions (11.1%) [19].

High VL (≥ 5000 copies/ml) at second-line HIV treatment initiation increased the odds of treatment failure (odds ratio (OR) 5.67; 95% CI: 3.40–9.45; P<0.0001). Patients who experienced virologic failure with first-line therapy and switched to second-line therapy after 12 months were more likely to experience a further increase in VL as a potential indicator for second-line therapy failure [77]. Several studies indicated that second-line therapy failure was associated with higher baseline VL measurements [26, 78–84]. Patients who had an experience of suboptimal adherence to second-line therapy were more likely to develop treatment failure compared to patients with optimal adherence (OR 1.92; 95% CI: 1.28, 2.86; P = 0.0013). Several published reports explained a relationship between suboptimal adherence to second-line therapy and the increase in failure rates [6, 22, 26, 71, 79, 81, 85–88]. In addition, patients with histories of suboptimal adherence to first-line therapy were also more likely to have suboptimal adherence to second-line therapy [50, 89]. It could increase the odds of second-line HIV treatment failure rate. This increased failure rate may also be linked to poor treatment adherence resulting from the more frequent toxicities associated with second-line ART regimens [65].

An advanced clinical stage (stage III or IV) of HIV at the commencement of second-line therapy increased the odds of treatment failure (OR 3.27; 95% CI: 2.07–5.19; P<0.0001). With this, baseline CD4 cell counts of < 100 cells/ml were linked to increased odds of treatment failure (OR 2.80; 95% CI: 1.83–4.29; P<0.0001). Growing evidence relate the advanced HIV and lower peak CD4 cell counts at baseline to the increased rates of failure with second-line therapy [6, 69, 77, 87, 90].

Some of the included studies also reported that patients with lengthy delays in initiating second-line therapy [25]; who were underweight [22]; who were on tuberculosis co-treatment [45]; who had depressive symptoms [23]; who were with practice of herbal or traditional medicine use [23]; and who were at younger age [53] had increased rates of treatment failure [63, 71, 77, 80, 90]. Although the relationship among depression, adherence and treatment failure is yet to be fully investigated, more than one-third of HIV-infected patients with depressive symptoms were found to have an elevated HIV-RNA VL in South Africa [91]. Patients with higher depression rating scales, with higher HIV-RNA VL and at a younger age were indicated to have increased patterns of ART missed doses [92]. A high probability of suboptimal adherence to ART among alcohol users was also reported [93] which can contribute to the ART failure. Despite widespread concern about concurrent traditional medicine use and ART, yet there is no sufficient evidence of whether traditional medicine use results in adverse effects or interactions that could limit the effectiveness of the ART or not [94]. With regard to HIV-tuberculosis co-treatment, suboptimal adherence to treatments was explained by a study report [95] and this interpretation could be related to the outcome of ART.

Contrary to other studies, one of the included studies reports that obese or overweight patients had a reduced proportion of failure to second-line ART [53]. Obese HIV patients were found to have higher CD4 counts compared to normal-weight patients [96]. A higher plasma concentration of second-line regimen containing darunavir-boosted with ritonavir was revealed in obese patients [97]. The WHO recommended second-line therapies for HIV-infection involve mainly ritonavir-boosted PI-based regimen. The ritonavir inhibits cytochrome P450 enzymes to which many of the medications are substrate [98]. The ritonavir-enzyme interaction can increase the plasma concentration of second-line therapy thereby protects treatment failure experience [99]. Although the relationship between elevated total bilirubin and second-line therapy failure is not fully clear, up to one-third of patients treated with atazanavir had elevated bilirubin as a marker of hepatotoxicity [100, 101]. Aligned with this, a higher discontinuation rate of ritonavir-boosted atazanavir [101] and super-boosting of lopinavir-ritonavir were linked with co-administration of a medication that inhibits hepatic cytochrome P450 enzymes [102].

Although the overall sample size was large enough, there are some limitations to note. First, the majority of data were derived from observational studies which resulted in a high degree of heterogeneity and a range of potential biases. As a result, we have used a random-effects model which is more appropriate in such anticipated heterogeneity. A series of subgroup analyses were also considered to reduce the degree of heterogeneity and presented them in percentages to indicate the extent of differences. Second, other additional potential explanations for second-line therapy failures including medication toxicities and drug-drug interactions might not have been adequately addressed. Third, we have included articles published only in the English language and this could under-or over-estimate the pooled proportion of second-line therapy failure in the SSA. Finally, the reporting of some variables pertaining to clinical and programmatic follow-ups were inconsistent, limiting the conclusiveness of the pooled factors associated with the second-line HIV treatment failure.

## Conclusion

The pooled proportion of second-line HIV treatment failure experienced by HIV-infected patients in SSA was found to be high. More common failure rates occurred at a 12–18 month period of follow-up after second-line therapy start, in children, and in the southern region of the SSA. Suboptimal adherence to second-line ART, higher HIV-RNA VL at baseline, lower peak values for CD4 cells, and advanced WHO clinical stages were among the key factors that have accelerated second-line HIV treatment failure in the setting. With this, prolonged delays in switching prior therapy, tuberculosis co-treatment, and other patient factors including younger age, depressive symptoms, underweight, and traditional medicine use were linked with the occurrence of second-line treatment failure. Therefore, optimal second-line HIV treatment approaches should critically consider immediate and aggressive VL suppression, rapid immune recovery, and excellent adherence to the therapy. Together with these approaches, more frequent clinical follow-ups and VL monitoring are recommended for the HIV-infected patients in SSA that help in rapid identification and intervention of failure cases. Finally, countries in the SSA should develop strategies and guidelines related to containment of second-line HIV treatment including intensive adherence support and intervention as routine clinical practice especially for patients with slow response to the therapies.

## Supporting information

S1 Table Completed PRISMA 2009 checklist. (DOC)

S2 Table Definition and month of treatment failure report for the included studies. (DOCX)

S3 Table Proportion of patients experiencing treatment failure in different subgroups. (DOCX)

## Abbreviations

AIDS: Acquired Immunodeficiency Syndrome
ART: Antiretroviral Therapy
CI: Confidence Interval
ES: Effect Size
HIV: Human Immunodeficiency Virus
JBI: Joanna Briggs Institute
OR: Odds Ratio
PRISMA: Preferred Reporting Items for Systematic review and Meta-analysis
RNA: Ribonucleic Acid
SSA: sub-Saharan Africa
VL: Viral Load
WHO: World Health Organization
