# Correction: Second-line HIV treatment failure in sub-Saharan Africa: A systematic review and meta-analysis

**DOI:** 10.1371/journal.pone.0223158

**Published:** 2019-09-24

**Authors:** The PLOS ONE Staff

Incorrect versions of Figs 2 and 3 were published in error, resulting in figures that are illegible. The publisher apologizes for the error. The authors have provided updated figure files. Please see an updated Fig 2 here.

**Fig 2:**
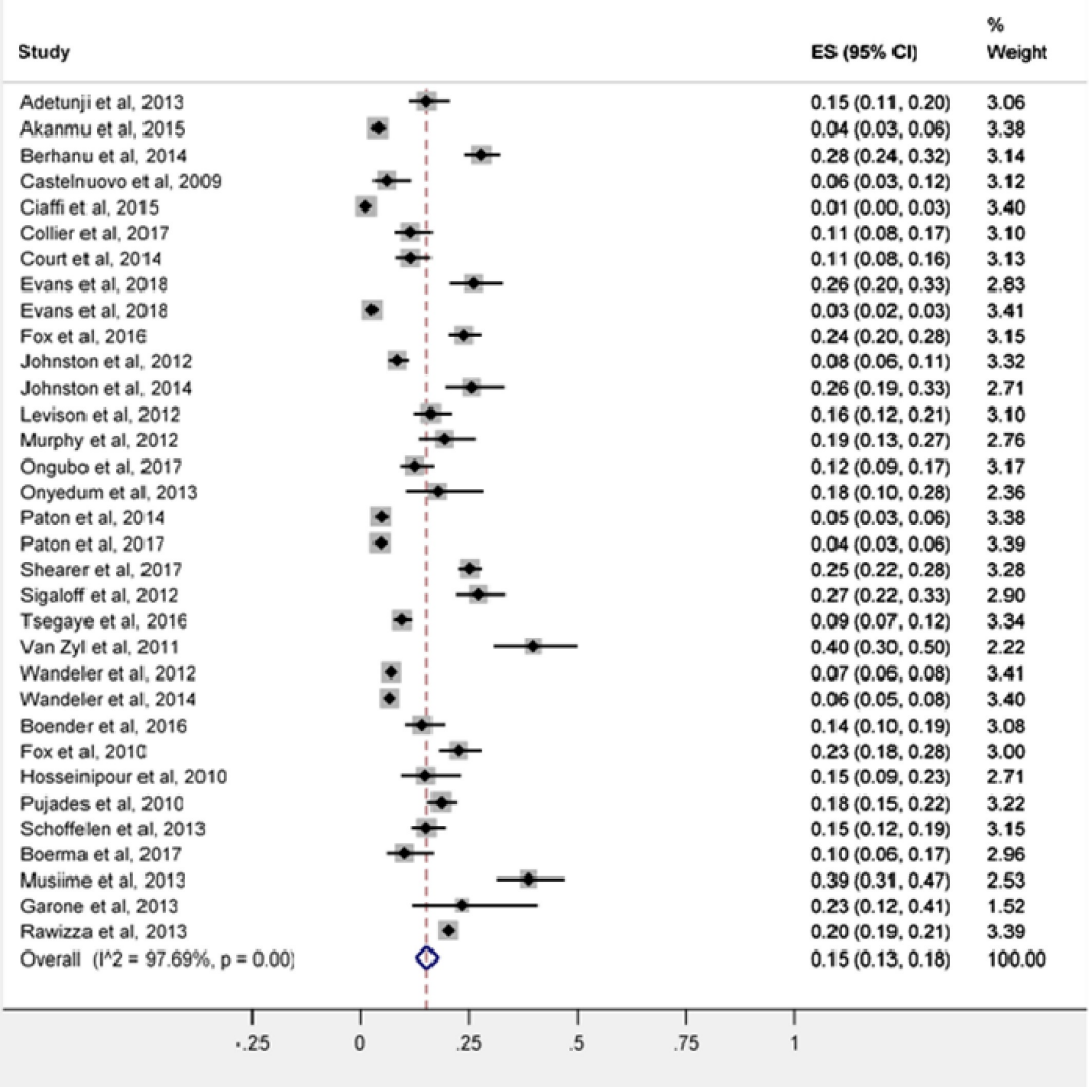
Forest pilot of proportion for second-line HIV treatment failure in SSA.

In addition, the updated, individual Fig 3 panels a, b, c, d are provided here as Supporting Information (S1 File).

## Supporting information

S1 File Updated, individual Fig 3 panels. (ZIP)
