# Supplementary material for: Second-line HIV treatment failure in sub-Saharan Africa: A systematic review and meta-analysis
Source: PLoS One. 2019 Jul 29;14(7):e0220159. doi: 10.1371/journal.pone.0220159 (PMC6663009; doi:10.1371/journal.pone.0220159)
Supplement: S2 Table — (DOCX) [file pone.0220159.s002.docx]

**S2 Table.** Definition and month of treatment failure report for the included studies

| **Name** | **Number of TF** | **Sample size** | **Median follow-up period** | **Definition of TF the studies had employed** |
| --- | --- | --- | --- | --- |
| Adetunji et al, 2013 | 34 | 225 | 12 months | VL > 400 copies/ml |
| Akanmu et al, 2015 | 25 | 318 | 24 months | VL > 400 copies/ml |
| Berhanu et al, 2014 | 129 | 372 | 6 months | VL >1000 copies/ml |
| Boender et al, 2016 | 32 | 227 | 12 months | VL>1000 copies/ml |
| Boerma et al, 2017 | 12 | 60 | 24 months | VL>1000 copies/ml |
| Castelnuovo et al, 2009 | 7 | 40 | 36 months | VL > 400 copies/ml |
| Ciaffi et al, 2015 | 5 | 451 | 12 months | VL>1000 copies/ml |
| Collier et al, 2017 | 23 | 101 | 6 months | VL>1000 copies/ml |
| Court et al, 2014 | 26 | 228 | 12 months | VL>1000 copies/ml |
| Evans et al, 2018 | 50 | 128 | 18 months | VL > 400 copies/ml |
| Evans et al, 2018 | 36 | 719 | 24 months | VL>1000 copies/ml |
| Fox et al, 2010 | 59 | 262 | 12 months | VL > 400 copies/ml |
| Fox et al, 2016 | 106 | 388 | 20 months | VL > 400 copies/ml |
| Garone et al, 2013 | 7 | 40 | 9 months | VL > 1000 copies/ml |
| Hosseinipour et al, 2010 | 15 | 101 | 12 months | VL > 400 copies/ml |
| Johnston et al, 2012 | 43 | 417 | 15 months | VL > 400 copies/ml |
| Johnston et al, 2014 | 39 | 122 | 15 months | VL > 400 copies/ml |
| Levison et al, 2012 | 43 | 322 | 10 months | VL > 1000 copies/ml |
| Murphy et al, 2012 | 26 | 136 | 12 months | VL > 1000 copies/ml |
| Musiime et al, 2013 | 55 | 142 | 12 months | VL > 400 copies/ml |
| Ongubo et al, 2017 | 35 | 376 | 9 months | VL>1000 copies/ml |
| Onyedum et al, 2013 | 12 | 68 | 12 months | VL > 1000 copies/ml |
| Paton et al, 2014 | 35 | 379 | 24 months | VL > 1000 copies/ml |
| Paton et al, 2017 | 45 | 336 | 36 months | VL > 400 copies/ml |
| Pujades et al, 2010 | 91 | 493 | 12 months | New stage 3 or 4 clinical event OR CD4 cell count <100 cells/mm3 OR VL > 1000 copies/ml |
| Rawizza et al, 2013 | 673 | 6714 | 6 months | VL > 1000 copies/ml |
| Schoffelen et al, 2013 | 48 | 191 | 20 months | VL>1000 copies/ml |
| Shearer et al, 2017 | 233 | 927 | 12 months | VL > 400 copies/ml |
| Sigaloff et al, 2012 | 63 | 232 | 12 months | VL >400 copies/ml or died/lost to follow up or switched to third-line ART |
| Tsegaye et al, 2016 | 67 | 356 | 24 months | New/recurrent stage 3 or 4 clinical event OR CD4 cell count at or below baseline value or persistent CD4 levels below 100 cells/mm3 OR died/lost to follow-up |
| Van Zyl et al, 2011 | 37 | 93 |  | VL > 500 copies/ml |
| Wandeler et al, 2012 | 240 | 2330 | 18 months | Persistent CD4 counts <100 cells/mm3 |
| Wandeler et al, 2014 | 122 | 1256 | 18 months | VL>1000 copies/ml |
| Total | 2, 473 | 18, 550 |  |  |

TF, treatment failure; VL, viral load
