# Supplementary material for: Second-line HIV treatment failure in sub-Saharan Africa: A systematic review and meta-analysis
Source: PLoS One. 2019 Jul 29;14(7):e0220159. doi: 10.1371/journal.pone.0220159 (PMC6663009; doi:10.1371/journal.pone.0220159)
Supplement: S3 Table — (DOCX) [file pone.0220159.s003.docx]

**Table 3**. Proportion of patients experiencing treatment failure in different subgroups

| **Subgroup** | **Number of patients** | **Number experiencing TF** | **PYs of follow-up** | **TF per 100 PYs (95% CI)** | **P-values** | **Heterogeneity (I^2^)** |
| --- | --- | --- | --- | --- | --- | --- |
| Month since second-line therapy initiation  < 12 months  12 -18 months  > 18 months | 7, 452  8, 210  2, 888 | 758  1, 311  404 | 3, 937.3  10, 292.25  5, 758.9 | 17.0 (12.0-22.0)  19.0 (15.0-22.0)  9.0 (6.0-11.0) | P < 0.001  P < 0.001  P < 0.001 | 80.81%  97.58%  94.92% |
| Study participants  Children  Adults  Mixed age groups | 202  10,809  7, 539 | 67  1, 572  901 | 262  15, 427.15  4, 299.3 | 19.0 (14.0-23.0)  14.0 (12.0-16.0)  18.0 (16.0-21.0) | -  P < 0.001  P = 0.10 | -  97.13%  47.93% |
| Regions of SSA  Southern Africa  Eastern Africa  Western Africa  Other SSA* | 6, 776  1, 411  7, 776  2, 587 | 1, 145  236  749  343 | 9, 172.45  2, 485  4, 737  3, 594 | 18.0 (14.0-23.0)  13.0 (8.0-18.0)  11.0 (2.0-20.0)  14.0 (6.0-19.0) | P < 0.001  P < 0.001  P < 0.001  P < 0.001 | 97.89%  93.64%  99.25%  96.37% |
| Second-line ART regimen  PI-based  Ritonavir boosted PI-based | 12, 250  6, 300 | 1, 608  865 | 11, 062.55  8, 925.9 | 16.0 (11.0-20.0)  14.0 (12.0-17.0) | P < 0.001  P < 0.001 | 96.43%  96.32% |

Asterisk (*), indicates mixed countries in different regions of SSA; PYs, person-years; TF, treatment failure; I^2^, chi-squared statistic showing degree of heterogeneity.
