# Supplementary figures and images for: Correction: Second-line HIV treatment failure in sub-Saharan Africa: A systematic review and meta-analysis
Source: PLoS One. 2019 Sep 24;14(9):e0223158. doi: 10.1371/journal.pone.0223158 (PMC6759147; doi:10.1371/journal.pone.0223158)

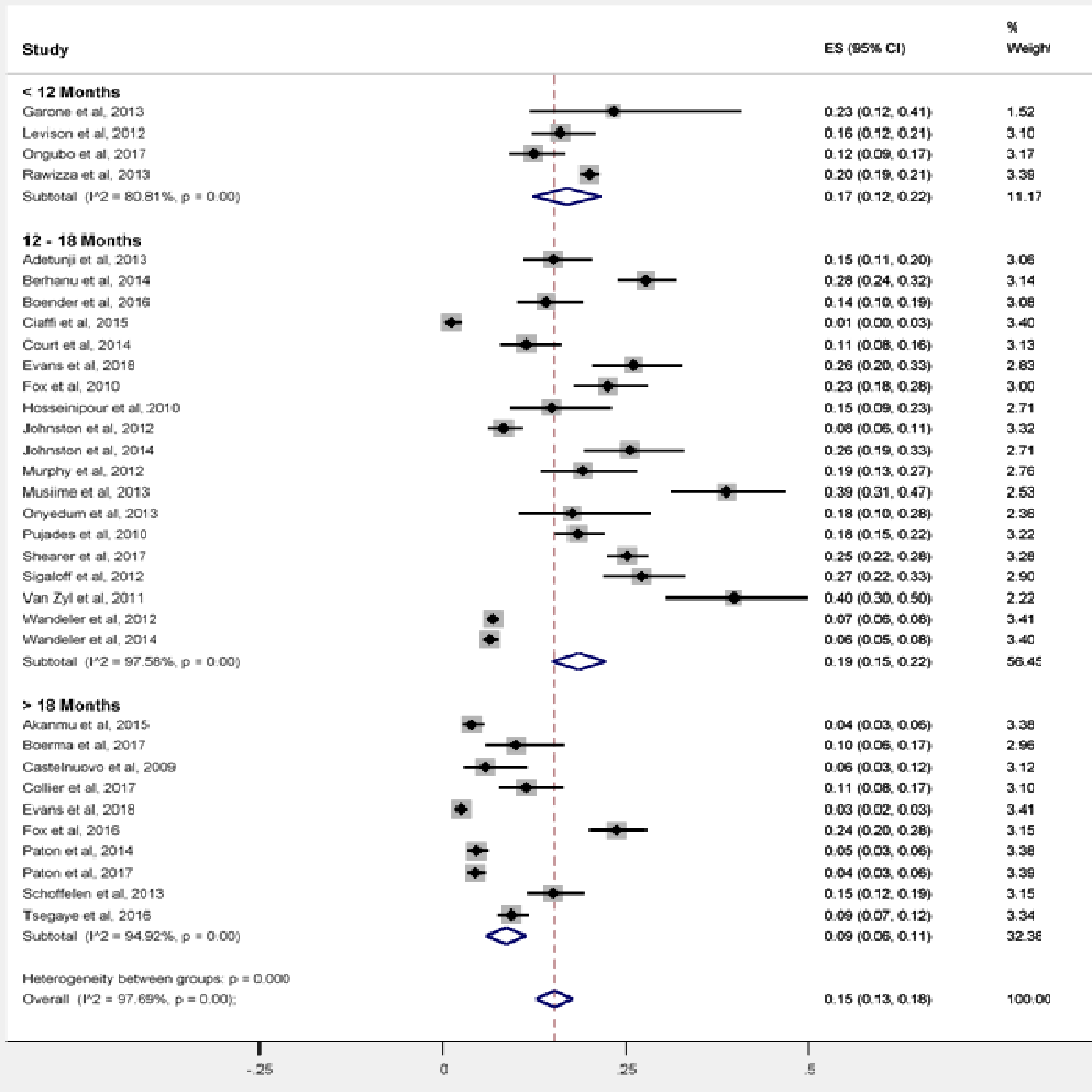

Supplement: S1 File — (ZIP) [file pone.0223158.s001.zip › Fig 3a.tif]

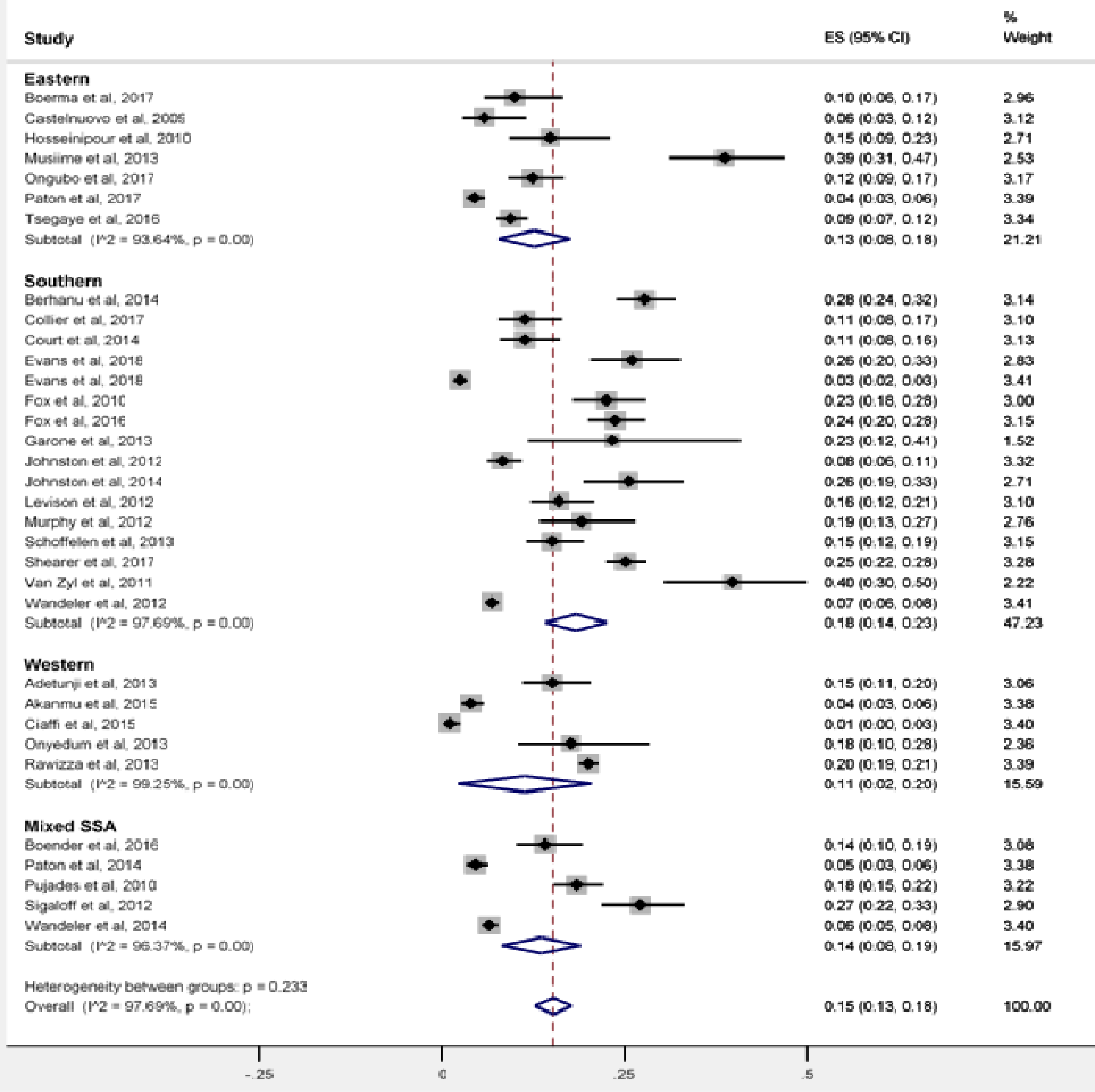

Supplement: S1 File — (ZIP) [file pone.0223158.s001.zip › Fig 3b.tif]

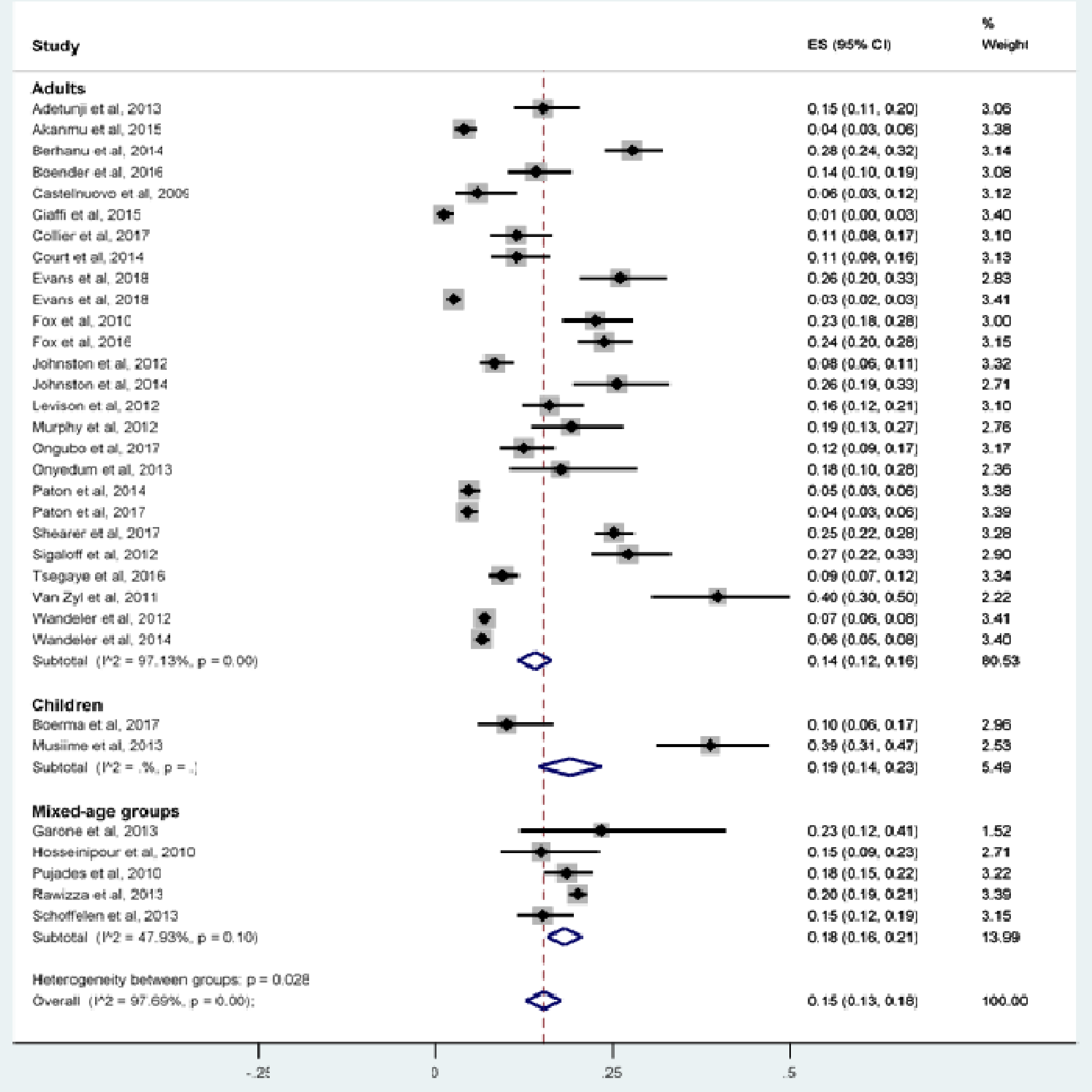

Supplement: S1 File — (ZIP) [file pone.0223158.s001.zip › Fig 3c.tif]

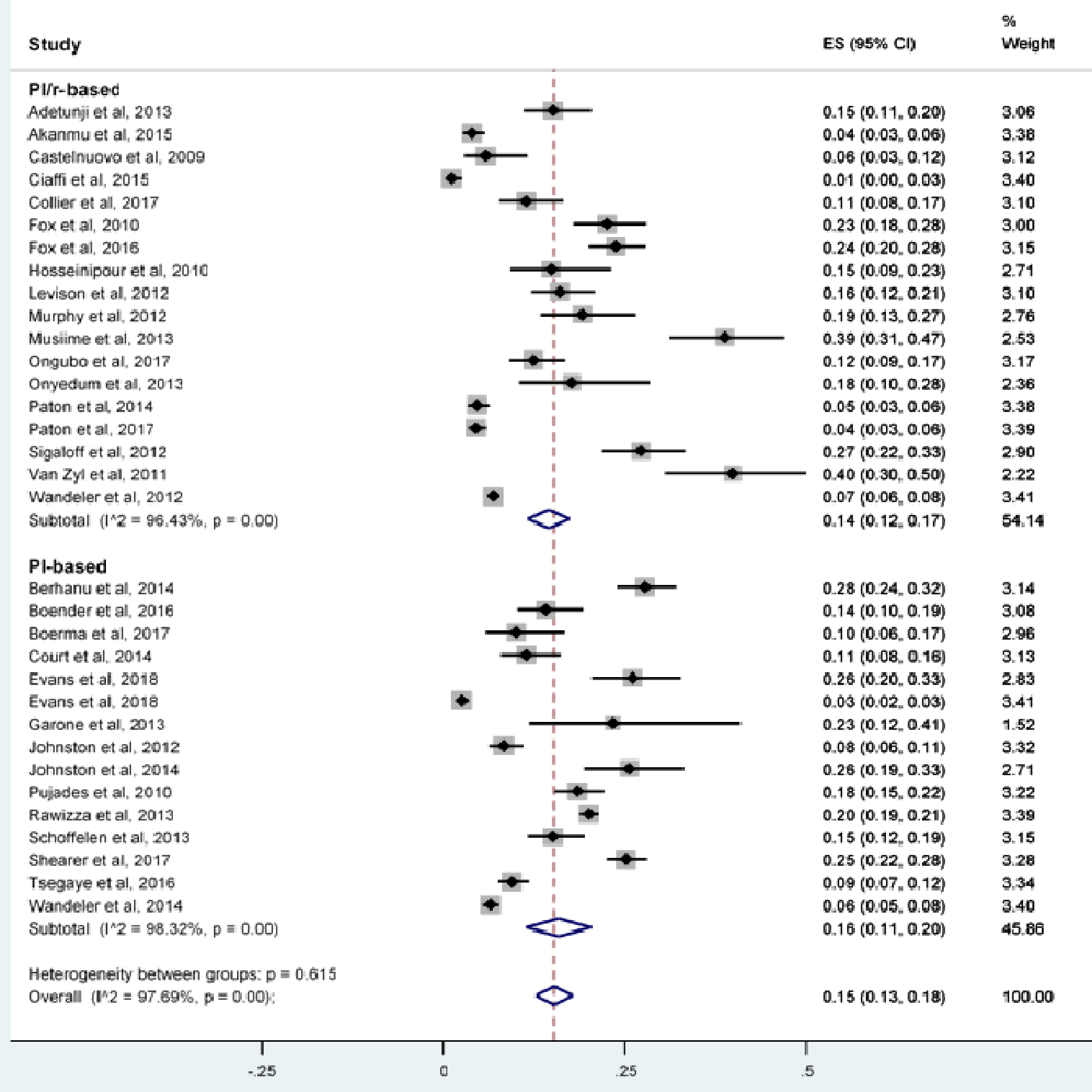

Supplement: S1 File — (ZIP) [file pone.0223158.s001.zip › Fig 3d.tif]
